# Involvement of HECTD1 in LPS-induced astrocyte activation via σ-1R-JNK/p38-FOXJ2 axis

**DOI:** 10.1186/s13578-021-00572-x

**Published:** 2021-03-30

**Authors:** Ying Tang, Mengchun Zhou, Rongrong Huang, Ling Shen, Li Yang, Zhongqiu Zhou, Hui Ren, Ying Bai

**Affiliations:** 1 Department of Pharmacology, School of Medicine, Southeast University, Nanjing, 210009 Jiangsu China; 2 Department of Pharmacy, Zhejiang Pharmaceutical College, Ningbo, Zhejiang China; 3 Jiangsu Provincial Key Laboratory of Critical Care Medicine, Department of Critical Care Medicine, Zhongda Hospital, School of Medicine, Southeast University, Nanjing, China

**Keywords:** Astrocyte, Activation, HECTD1, LPS, Neuroinflammation

## Abstract

**Background:**

Astrocytes participate in innate inflammatory responses within the mammalian central nervous system (CNS). HECT domain E3 ubiquitin protein ligase 1 (HECTD1) functions during microglial activation, suggesting a connection with neuroinflammation. However, the potential role of HECTD1 in astrocytes remains largely unknown.

**Results:**

Here, we demonstrated that HECTD1 was upregulated in primary mouse astrocytes after 100 ng/ml lipopolysaccharide (LPS) treatment. Genetic knockdown of HECTD1 in vitro or astrocyte-specific knockdown of HECTD1 in vivo suppressed LPS-induced astrocyte activation, whereas overexpression of HECTD1 in vitro facilitated LPS-induced astrocyte activation. Mechanistically, we established that LPS activated σ-1R-JNK/p38 pathway, and σ-1R antagonist BD1047, JNK inhibitor SP600125, or p38 inhibitor SB203580 reversed LPS-induced expression of HECTD1, thus restored LPS-induced astrocyte activation. In addition, FOXJ2 functioned as a transcription factor of HECTD1, and pretreatment of primary mouse astrocytes with BD1047, SB203580, and SP600125 significantly inhibited LPS-mediated translocation of FOXJ2 into the nucleus.

**Conclusions:**

Overall, our present findings suggest that HECTD1 participates in LPS-induced astrocyte activation by activation of σ-1R-JNK/p38-FOXJ2 pathway and provide a potential therapeutic strategy for neuroinflammation induced by LPS or any other neuroinflammatory disorders.

**Supplementary Information:**

The online version contains supplementary material available at 10.1186/s13578-021-00572-x.

## Background

Inflammatory responses are associated with almost all CNS insults, including infection, acute trauma, depression, and chronic neurodegenerative diseases [1]. As the most abundant CNS cell type, astrocytes are able to respond to a vast array of CNS insults and become activated with elevated expression of GFAP [2–4]. Increasing evidences show the key role of astrocytes in neuroinflammation and activated astrocytes secrete a number of inflammatory cytokines and mediators affecting nearby cells of all types in many ways [5, 6]. What’s more, astrocytes can promote leukocyte extravasation and help to recruit leukocytes into CNS parenchyma, which further aggravates central inflammatory reaction [1]. Given the many ways in which astrocytes can interact with other inflammatory cells, there is increasing interest in the influence of astrocytes on inflammation in diverse CNS disorders.

HECTD1 is a HECT domain E3 ubiquitin ligase and is widely expressed in a range of human and murine primary cells and cell lines [7], including GBM (glioblastoma multiforme) cell line LN-229 [8], macrophages [9], and insulin secreting β-cells [10]. Previous studies have showed an important role of HECTD1 in the regulation of Wnt signaling and development of neural tube [11]. Also, the cranial mesenchyme cell migration is correlated with HECTD1 expression [11, 12]. Furthermore, interaction of HECTD1 with ribosomal protein subunit 3 contributes to degradation of IκBα, and subsequently facilitates inflammatory response [13]. Recent study demonstrated that HECTD1 is involved in regulation of microglia activation by promoting HSP90 degradation via ubiquitination, which implies the correlation between HECTD1 and inflammation in the CNS [14]. Although the studies of HECTD1 in multiple cells have been conducted [8], the role of HECTD1 in astrocyte activation currently remains elusive [13].

LPS, a bacterial endotoxin, was applied to induce astrocyte activation modeling the neuroinflammation. In this study, we applied 100 ng/ml LPS to induce inflammatory responses in primary mouse astrocytes. LPS significantly increased the expression of HECTD1, and genetic knockdown of HECTD1 inhibited LPS-induced astrocyte activation, whereas overexpression of HECTD1 exacerbated LPS-induced astrocyte activation. In addition, our study showed LPS upregulated HECTD1 expression via σ-1R-JNK/p38-FOXJ2 pathway, resulting in astrocyte activation. These findings reveal a novel function of HECTD1 in astrocyte activation induced by LPS.

## Materials and methods

### Animals

All animal procedures were conducted according to protocols approved by the Institutional Animal Care and Use Committee of the Medical School of Southeast University. Adult male (6–8 weeks) C57BL/6 J mice (GemPharmatech, Nanjing, China) kept under a constant temperature and 12:12 h light: dark cycles were used. HECTD1^flox/flox^ mice (HECTD1^f/f^, C57BL/6 J background) were generated and maintained at GemPharmatech (Nanjing, China). Food and water were provided a*d libitum*.

### Reagents

LPS (#L2630) were obtained from Sigma-Aldrich. SP600125, BD1047 and SB203580 were obtained from Calbiochem (San Diego, CA). The concentrations of these inhibitors were based on the concentration-curve study and our previous reports [15, 16].

### Recombinant virus construction and stereotaxic injection

The adeno-associated virus (AAV), HBAAV2/9-GFAP-cre-T2A-EGFP, was constructed and synthesized by HANBIO (1.2 × 10^12^ viral genome/µl, Shanghai, China). The stereotaxic injection site was as follows: 0.3 mm behind the bregma and 1.0 mm lateral from the sagittal midline, at a depth of 2.2 mm from the skull surface. After injection, the needle was left in place for 10 min to ensure well distribution of the virus.

### Cell cultures

Primary mouse astrocytes were obtained from postnatal (P1-P2) C57BL/6 J mice. Briefly, whole brains were removed quickly and kept in cold phosphate-buffered serum (PBS; Gibco, #10,010,001). Then membranes and large blood vessels were removed mechanically using gauze. The dissected brain cortices were placed in medium supplemented with PBS and digested with trypsin–EDTA (Gibco, #25,200,056). Subsequently, the cells were planted on poly-L-lysine precoated cell culture flasks containing DMEM (Corning, #32,016,001) supplemented with fetal bovine serum (FBS, 10% v/v) and penicillin/streptomycin (1% v/v). The cultures were maintained in a humidified chamber (37 °C, 5% CO_2_ incubator). After 7 to 10 days, cells were harvested by trypsinization.

### Western blot analysis

Tissue was rapidly dissected from HECTD1^f/f^ or wild type mice. Cells were treated with Nuclear and Cytoplasmic Extraction Kit (Beyotime, #P0028). Proteins were extracted in a RIPA lysis buffer (Beyotime, #P0013B), separated via sodium dodecyl sulfate–polyacrylamide gel electrophoresis and electrophoretically transferred onto polyvinylidene fluoride membranes. After blocking with 5% nonfat dry milk in Tris-buffered saline with Tween-20 (Aladdin, #T104863), the membranes were probed with antibodies overnight at 4 °C, followed by incubation with a horseradish peroxidase-conjugated goat anti-mouse (ZSGB-BIO, #ZB5305) or goat anti-rabbit (ZSGB-BIO, #ZB5301) IgG secondary antibody (1:2000). The antibodies were as follows: anti-GFAP (#G3893), obtained from Sigma-Aldrich; anti-σ-1R (#sc137075), purchased from Santa Cruz Biotechnology; anti-GAPDH (#60,004), anti-HECTD1 (#20,605–1-AP), obtained from Proteintech Group; anti-Histone H3 (#9715S), anti-p-ERK/ERK (#9106S/9107S), anti-p-JNK/JNK (#9251S/9252S), anti-p-Akt/Akt (#9271S/9272S), anti-p-p38/p38 (#9211S/9212S), obtained from Cell Signaling Technology; anti-FOXJ2 (#36,867), purchased from Signalway Antibody LLC. Detection was performed using a MicroChemi 4.2® (DNR, Israel) digital image scanner. The band intensity was quantified using ImageJ software (NIH).

### Chromatin immunoprecipitation assay (ChIP)

ChIP assays were performed according to the manufacturer’s protocol (Beyotime, #P2078). Chromatin solutions were sonicated and incubated with anti-FOXJ2 or with control IgG, and rotated overnight at 4 °C. DNA–protein cross-links were reversed and chromatin DNA was purified and subjected to PCR analysis. The forward primer (5′-TCACAGAGGATCCTTAAAGAGTGT-3) and reverse primer (5′-AGGACAGAGAGTTCCCACTGA-3′) were used to amplify the HECTD1 promoter sequence. After amplification, PCR products were resolved on a 2% agarose gel and visualized by ethidium bromide staining.

### MTT assays

Cell viability was measured by MTT assay. Briefly, cells were seeded in 96-well plates, and MTT dye was administrated 0.5 h before the termination of experiment. The absorbance was obtained using a Synergy H1 Multi-Mode Reader (BioTek, Winooski, VT, USA) with a wavelength of 570 nm.

### Immunofluorescence staining

Cells were cultured on cover-slips and treated with LPS for 12 h. Cells were fixed with 4% paraformaldehyde and then permeabilized with 0.3% Triton X-100 in PBS. After blocked with 10% normal goat serum in 0.3% Triton X-100, cells were incubated with mouse anti-GFAP antibodies (1:800; Sigma-Aldrich, #G3893) and/or rabbit anti-HECTD1 (1:200; Proteintech Group, #20,605–1-AP) overnight at 4 °C. Cells were subsequently incubated with the AlexaFluor 488-conjugated anti-rabbit IgG (1:250; Invitrogen, #A-11034) or Alexafluor 594-conjugated anti-mouse secondary antibody (1:250; Invitrogen, #A-11005). Immunofluorescence images were captured by microscopy (Olympus DP73, Olympus, Tokyo, Japan). The quantification of fluorescence intensity was performed using ImageJ software.

### Target DNA deletion/upregulation using CRISPR/ Cas9 technology

Primary mouse astrocytes were transiently transfected with the CRISPR/Cas9 plasmids according to the manufacturer’s recommended protocol (Santa Cruz) to delete/upregulate HECTD1. The transfection efficiency was determined by western blotting. In brief, cells were seeded in a 6-well plate (2 × 10^5^/well) with 2 ml of antibiotic-free standard growth medium and grew to 40–50% confluency. Then, 200 µl of the Plasmid DNA/UltraCruz® Transfection Reagent Complex, containing 2 µg of plasmid DNA and 10 µl of the UltraCruz® Transfection Reagent in Plasmid Transfection Medium, was added dropwise into each well. Finally, gentle mixing was performed by swirling the plate, and the cells were incubated for 24 h under normal conditions prior to subsequent experiments.

### Statistical analyses

The data are presented as the mean ± standard error of the mean (SEM). Significance was established using a t-test for paired values. One/Two-way ANOVA followed by Holm-Sidak post hoc test was used for comparisons of 3 or more groups (multi-group comparison). *p*-value of *p* < 0.05 was regarded as statistically significant. Statistical analysis was performed using GraphPad Prism 8.0.2 Software.

## Results

### LPS increases HECTD1 expression in primary mouse astrocytes

**Fig. 1:**
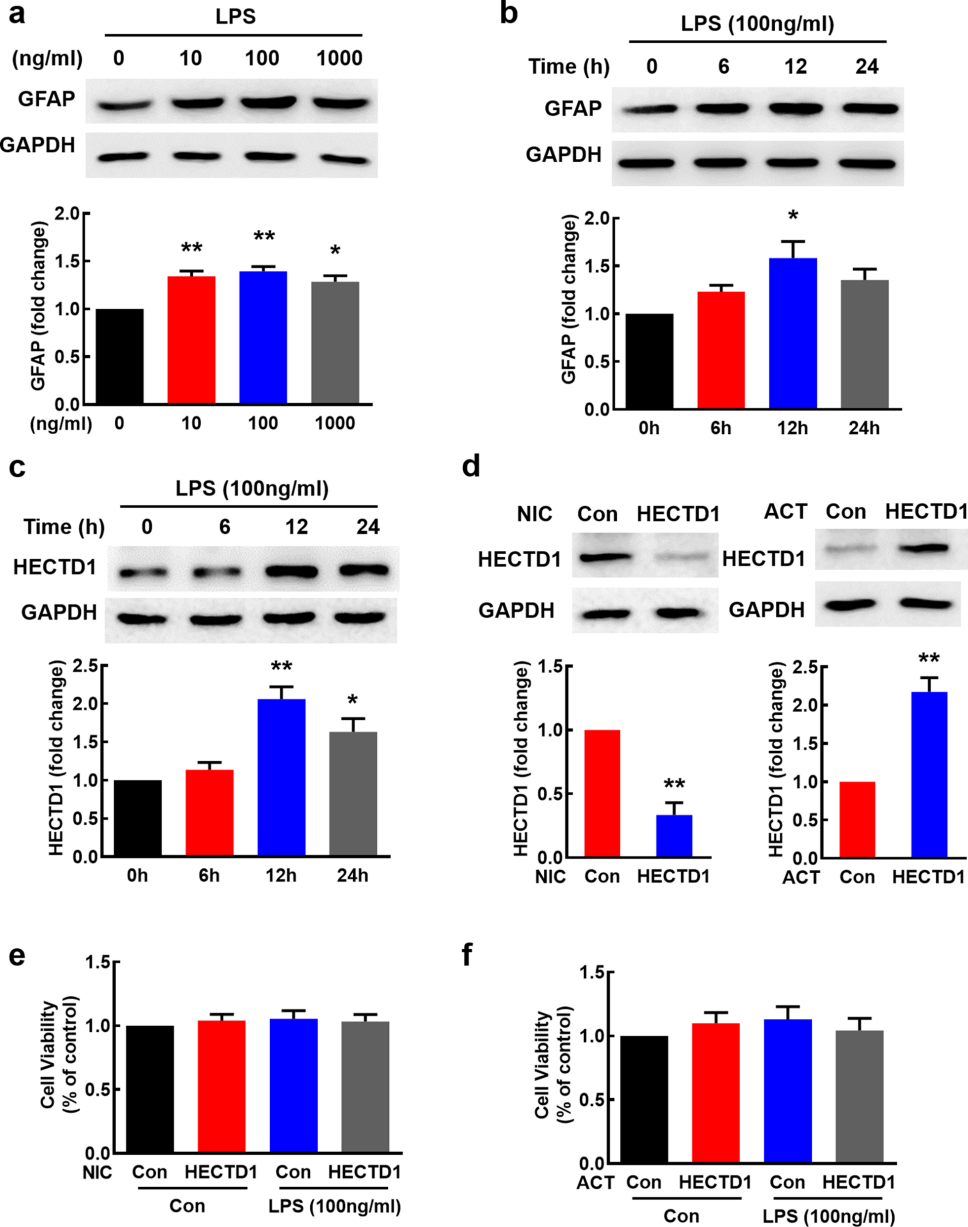
LPS induces astrocyte activation and HECTD1 upregulation in primary mouse astrocytes. **a** Treatment of astrocytes with different concentration of LPS (10 ng/ml, 100 ng/ml, 1 μg/ml) for 12 h significantly increased astrocyte activation as determined by GFAP expression. *p < 0.05 and **p < 0.01 *vs*. the 0 ng/ml group. **b**, **c** Treatment of primary mouse astrocytes with 100 ng/ml LPS significantly increased the expression of GFAP (**b**) and HECTD1 (**c**) in a time-dependent manner. *p < 0.05 and **p < 0.01 *vs*. the 0 h group. **d** Transfection with HECTD1 NIC downregulated HECTD1 expression and HECTD1 ACT upregulated HECTD1 expression in primary mouse astrocytes. Cells were transfected with HECTD1 NIC or ACT for 24 h followed by measurement of HECTD1 expression. Representative immunoblots and the densitometric analysis from three separate experiments are presented. **p < 0.01 *vs*. the control group. **e**, **f** MTT assay showed the effect of HECTD1 NIC and ACT transfection with/without 100 ng/ml LPS treatment on the viability of primary mouse astrocytes. All the data are presented as mean ± SEM of three individual experiments

According to our previous study, LPS induced astrocyte activation and increased the expression of GFAP in vitro [17]. As shown in Fig. 1a, astrocytes treated with varying concentrations of LPS resulted in GFAP upregulation with a peak expression at 100 ng/ml. To further investigate the effect of LPS on astrocyte, we exposed primary mouse astrocytes to 100 ng/ml and 1 μg/ml LPS for varying time points. Compared with the control group, GFAP was significantly upregulated at 12 h, as shown in Fig. 1b and Additional file 1: Fig. S1a. The two concentrations of LPS that induced astrocyte activation changed the expression of HECTD1 with extremely different trends. 100 ng/ml LPS upregulated the expression of HECTD1 with the peak response at 12 h (Fig. 1c), while 1 μg/ml LPS downregulated the expression of HECTD1 (Additional file 1: Fig. S1b). To investigate the role of HECTD1 in astrocyte activation, we transfected primary mouse astrocytes with the HECTD1 CRISPR double nickase plasmid (NIC) or CRISPR activation plasmid (ACT). As shown in Fig. 1d, HECTD1 NIC downregulated the expression of HECTD1 and HECTD1 ACT upregulated the expression of HECTD1. Furthermore, HECTD1 NIC and ACT didn’t have a significant impact on cell viability (Fig. 1e, f).

### HECTD1 regulates astrocyte activation induced by LPS

**Fig. 2:**
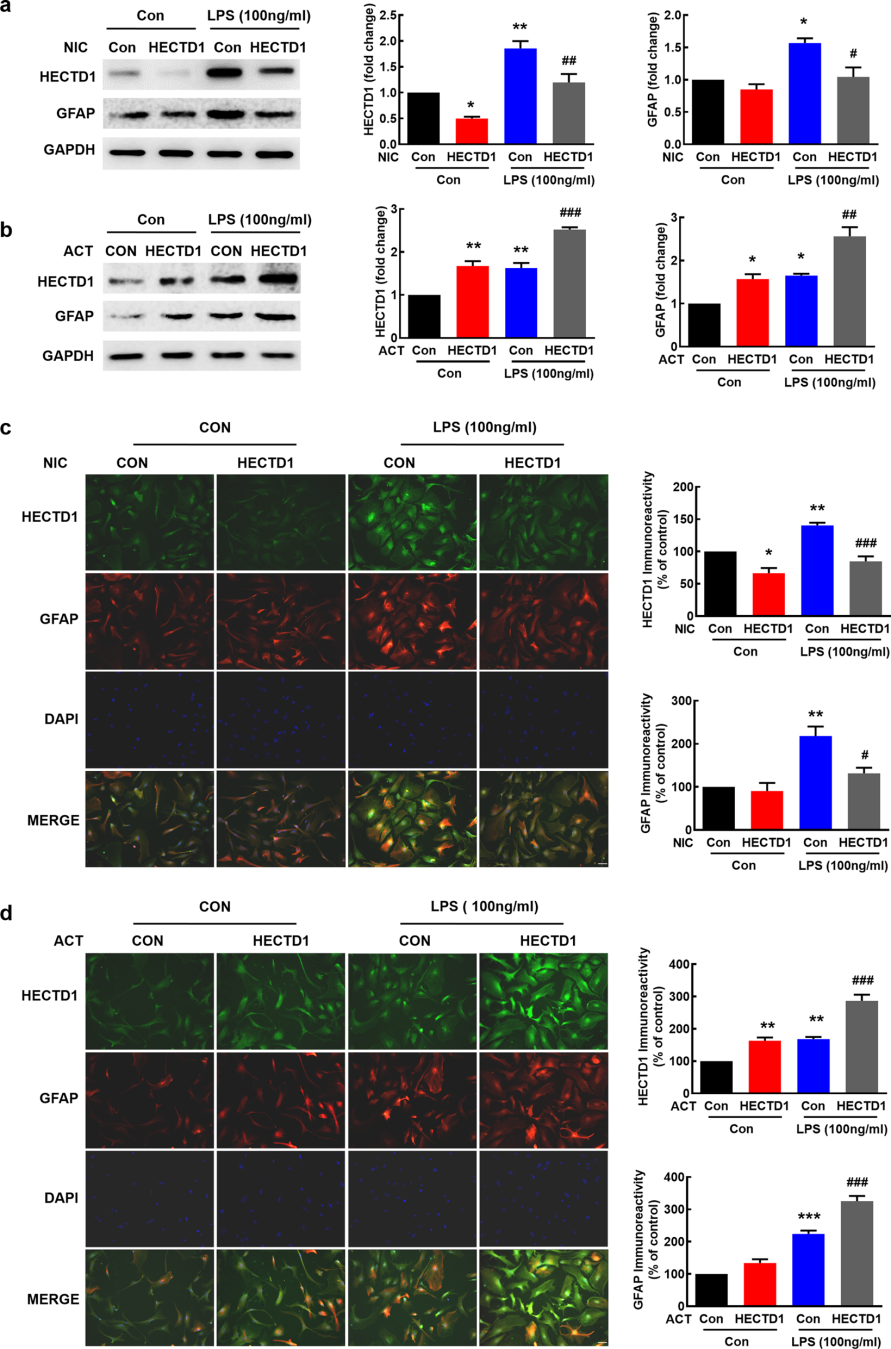
Role of HECTD1 in astrocyte activation induced by LPS in vitro. **a**, **b** Representative immunoblots and the densitometric analysis from three separate experiments showed the effect of HECTD1 NIC and ACT transfection with/without 100 ng/ml LPS treatment on GFAP and HECTD1 expression. Transfection with HECTD1 NIC inhibited LPS-induced GFAP expression (**a**). Transfection with HECTD1 ACT enhanced LPS-induced GFAP expression (**b**). **c**, **d** Representative images of HECTD1 fluorescence (green) and GFAP fluorescence (red) showed the effect of HECTD1 NIC and ACT transfection with/without 100 ng/ml LPS treatment on GFAP and HECTD1 expression. Scale bar = 50 μm. Quantification of GFAP and HECTD1 immunofluorescence intensity used Image J software. *p < 0.05, **p < 0.01, and ***p < 0.001 *vs*. the control group; #*p* < 0.05, ##*p* < 0.01, and ###*p* < 0.001 *vs*. the LPS-treated control group

Next, we dissected the role of HECTD1 in astrocyte activation induced by 100 ng/ml or 1 μg/ml LPS. Transfection with HECTD1 NIC significantly reversed the increased expression of HECTD1 and GFAP induced by 100 ng/ml LPS (Fig. 2a), while HECTD1 NIC further promoted the upregulation of GFAP induced by 1 μg/ml LPS (Additional file 1: Fig. S1c). Transfection with HECTD1 ACT notably increased the upregulation of GFAP induced by 100 ng/ml LPS (Fig. 2b), but reversed the decrease of HECTD1 and upregulation of GFAP induced by 1 μg/ml LPS (Additional file 1: Fig. S1d). These findings were further confirmed by immunostaining (Fig. 2c, d and Additional file 1: Fig. S2). Taken together, these findings suggested that HECTD1 played a critical role in LPS-mediated astrocyte activation in vitro.

**Fig. 3:**
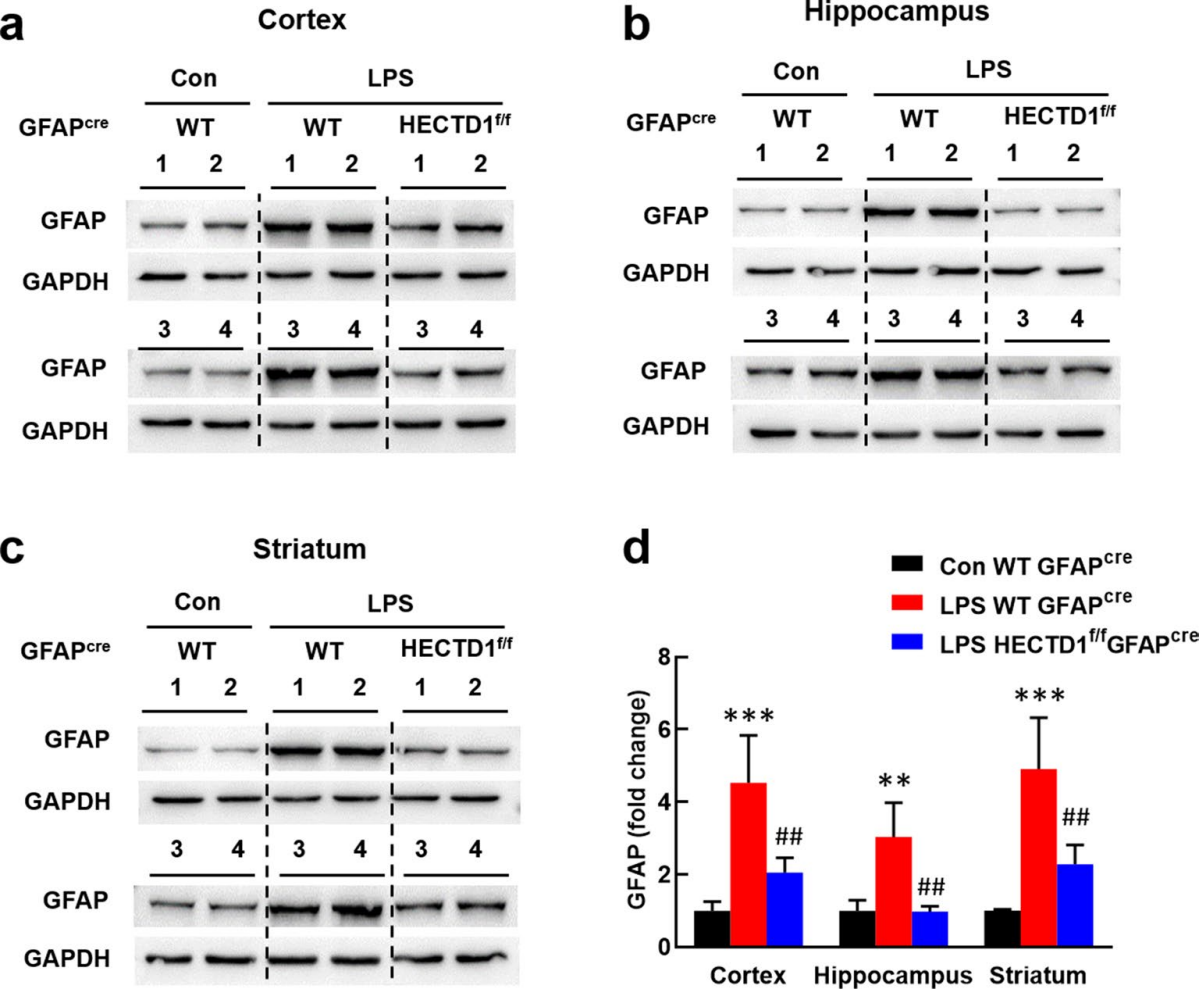
Role of HECTD1 in astrocyte activation induced by LPS in vivo. **a**-**c** HECTD1 deletion reversed LPS-induced increased expression of GFAP in cortex (**a**), hippocampus (**b**), and striatum (**c**). **d** Densitometric analysis of (**a**), (**b**), and (**c**). HECTD1^f/f^ mice or C57BL/6 J mice were infused with HBAAV2/9-GFAP-cre-T2A-EGFP into the lateral ventricle. Four weeks later animals were treated with LPS (i.p., 20 mg/kg) for 4 h and then sacrificed for western blot analysis. n = 4 animals for each group. **p < 0.01 and ***p < 0.001 *vs*. the Con + WT + GFAP^cre^ group; ##*p* < 0.01 *vs*. the LPS + WT + GFAP^cre^ group

Next, we explored the role of HECTD1 in LPS-induced astrocyte activation in vivo. We infused adeno-associated virus vector HBAAV2/9-GFAP-cre-T2A-EGFP into the lateral ventricle of HECTD1^f/f^ mice or C57BL/6 J mice. Four weeks later animals were treated with LPS (i.p., 20 mg/kg) for 4 h and then sacrificed to estimate the level of GFAP. Our results showed that HECTD1 deletion reversed LPS-induced increase of GFAP in cortex (Fig. 3a, d), hippocampus (Fig. 3b, d), and striatum (Fig. 3c, d), respectively.

### LPS upregulates σ-1R expression and activates the JNK/p38 and Akt pathway

**Fig. 4:**
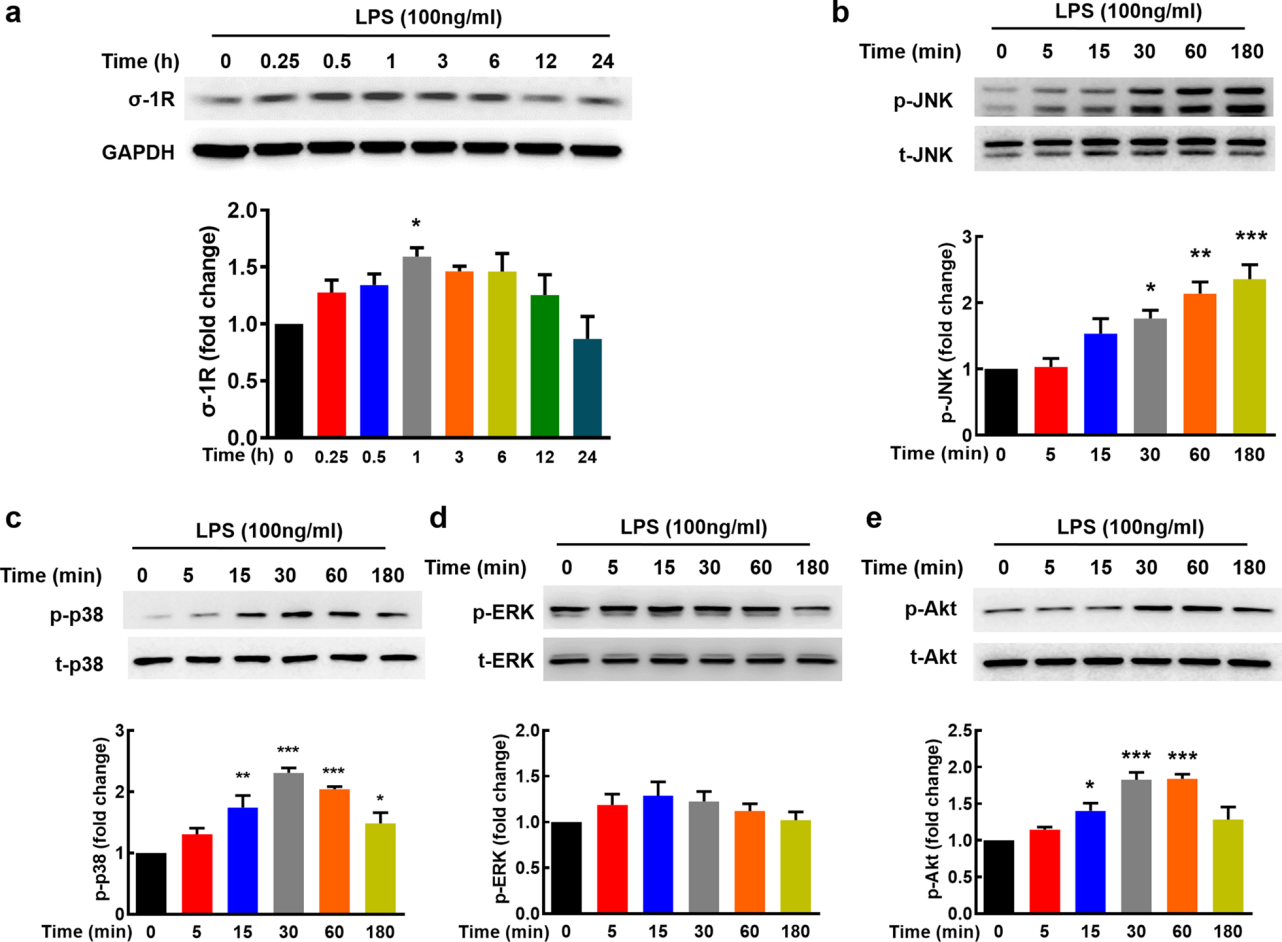
Upregulation of σ-1R and activation of JNK/p38 and Akt pathway in LPS-induced astrocyte activation. **a** Treatment of astrocytes with 100 ng/ml LPS significantly increased the expression of σ-1R. Representative immunoblots and the densitometric analysis from three separate experiments are presented. **b**-**e** Western blot analysis of time-dependent activation of JNK (**b**), p38 (**c**), ERK (**d**) and Akt (**e**) by LPS (100 ng/ml) in primary mouse astrocytes. All the data are presented as mean ± SEM of three individual experiments. *p < 0.05, **p < 0.01, and ***p < 0.001 *vs*. the control group

It has been shown previously that σ-1R participates in astrocyte activation induced by LPS [17]. Consistent with the previous study, 100 ng/ml LPS notably increased the expression of σ-1R (Fig. 4a). To explore the potential downstream mechanisms, we detected the MAPKs and Akt-mTOR pathways, two well-known pathways involved in regulation of inflammatory processes. Thus, we examined the effect of LPS on the activation of JNK, p38, ERK, and Akt. LPS treatment resulted in increased phosphorylation of JNK and p38 with peak responses at 180 and 30 min, respectively (Fig. 4b, c), but no significant effect on phosphorylation of ERK was observed (Fig. 4d). AKT phosphorylation was activated with a peak response at 60 min (Fig. 4e).

### Role of σ-1R, JNK/p38, and Akt in astrocyte activation induced by LPS

**Fig. 5:**
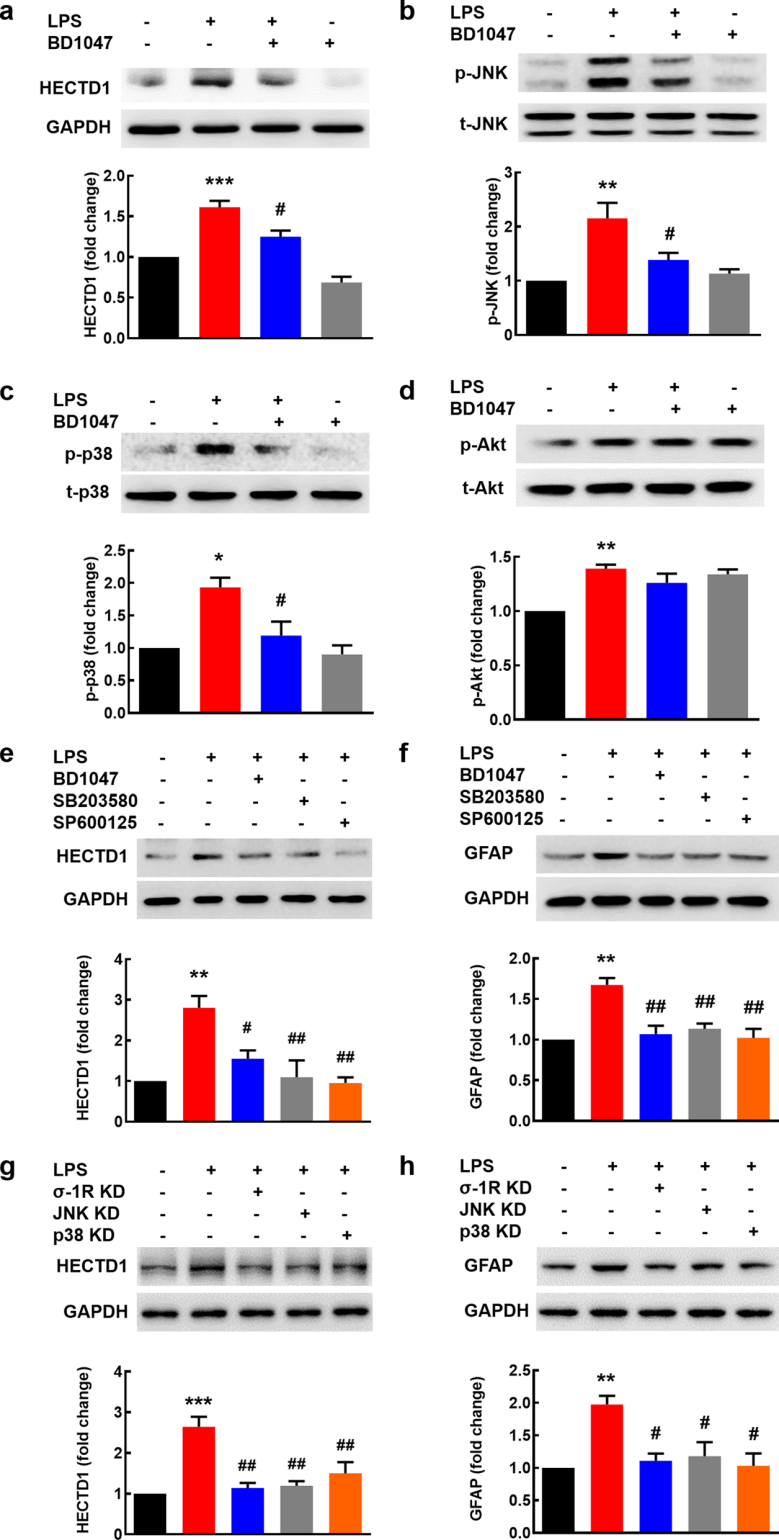
Engagement of σ-1R and JNK/p38 in LPS-induced HECTD1 upregulation. **a** Pretreatment of astrocytes with BD1047 inhibited LPS-induced increase of HECTD1 expression. **b** Pretreatment of astrocytes with BD1047 inhibited LPS-induced increase of p-JNK expression. **c** Pretreatment of astrocytes with BD1047 inhibited LPS-induced increase of p-p38 expression. **d** The effect of BD1047 on p-Akt expression post LPS. All the data are presented as mean ± SEM of three individual experiments. *p < 0.05, **p < 0.01, and ***p < 0.01 *vs*. the control group; #*p* < 0.05 *vs*. the LPS-treated control group. **e** Pretreatment of primary mouse astrocytes with BD1047, SB203580, and SP600125 resulted in inhibition of the LPS-mediated HECTD1 elevation. **f** Pretreatment of primary mouse astrocytes with BD1047, SB203580, or SP600125 resulted in inhibition of the LPS-mediated GFAP elevation. All the data are presented as mean ± SEM of three individual experiments. **p < 0.01 *vs*. the control group; #*p* < 0.05, ##*p* < 0.01 *vs*. the LPS-treated control group. **g** Pretreatment of primary mouse astrocytes with siRNA of σ-1R, JNK, or p38 resulted in inhibition of the LPS-mediated HECTD1 elevation. All the data are presented as mean ± SEM of three individual experiments. ***p < 0.001 vs. the control group; ##*p* < 0.01 vs. the LPS-treated control group. **h** Pretreatment of primary mouse astrocytes with siRNA of σ-1R, JNK, or p38 resulted in inhibition of the LPS-mediated GFAP elevation. All the data are presented as mean ± SEM of three individual experiments. **p < 0.01 vs. the control group; #*p* < 0.05 vs. the LPS-treated control group

According to our previous study, BD1047, an antagonist of σ-1R, weakened the increase of GFAP induced by LPS [15]. We thus sought to examine whether σ-1R also played a role in LPS-mediated upregulation of HECTD1, JNK/p38 and Akt. Astrocytes were pretreated with BD1047 for 1 h followed by incubation with LPS for 12 h, and then lysed for assessment of HECTD1 expression. Pretreatment of cells with BD1047 significantly reversed LPS-induced upregulation of HECTD1 (Fig. 5a). JNK and p38 phosphorylation was notably inhibited by pretreatment of BD1047 (Fig. 5b, c). However, BD1047 treatment didn’t lead to significant difference in phosphorylation of AKT comparing to LPS treatment group (Fig. 5d). To investigate whether σ-1R regulates HECTD1 and GFAP expression via JNK/p38 pathway, astrocyte was pretreated with the σ-1R antagonist BD1047, JNK inhibitor SP600125, or p38 inhibitor SB203580 for 1 h followed by treatment with LPS for 12 h. As shown in Fig. 5e, f, the increased expression of HECTD1 and GFAP induced by LPS was significantly inhibited by the pretreatment of BD1047, SP600125 or SB203580. To test the signaling pathway specificity via σ-1R-JNK/p38 axis, we used siRNA of σ-1R, JNK, and p38 to confirm the inhibitor results. The increased expression of HECTD1 and GFAP induced by LPS was significantly reversed by knockdown of σ-1R, JNK, or p38 (Fig. 5g, h). Taken together, these findings suggested that LPS induced astrocyte activation via σ-1R- JNK/p38-HECTD1 axis.

### FOXJ2 involves in LPS-induced upregulation of HECTD1

**Fig. 6:**
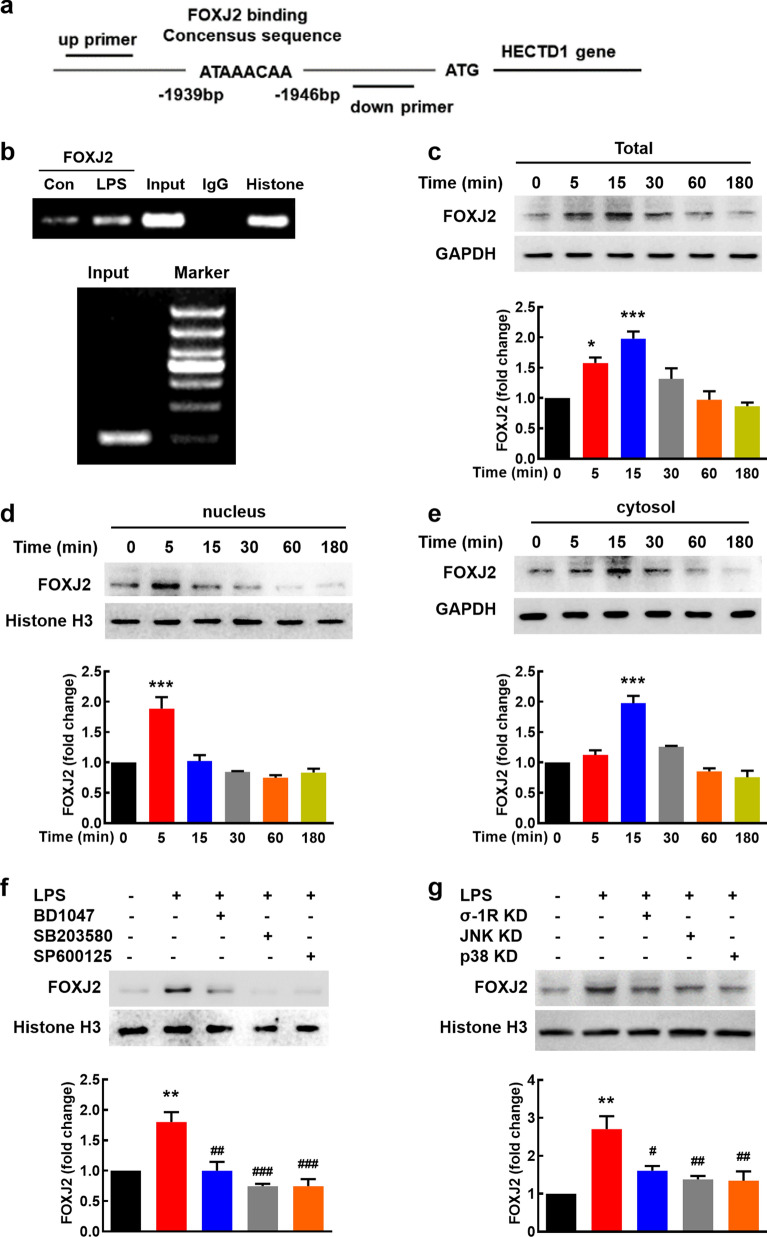
LPS-mediated induction of HECTD1 involves translocation of FOXJ2 into the nucleus. **a** Illustration of the consensus binding site of FOXJ2 to the HECTD1 promoter. **b** ChIP assay demonstrated LPS-mediated binding of FOXJ2 to the HECTD1 promoter. **c**-**e** Time-dependent effect of LPS on the total level of FOXJ2 in whole cells (**c**), nucleus (**d**), and cytosol (**e**). *p < 0.05 and ***p < 0.001 *vs*. the control group. **f** Pretreatment of primary mouse astrocytes with BD1047, SB203580, and SP600125 significantly inhibited LPS-mediated translocation of FOXJ2 into the nucleus. All the data are presented as mean ± SEM of three individual experiments. **p < 0.01 *vs*. the control group; ##*p* < 0.01 and ###*p* < 0.001 *vs*. the LPS-treated control group. **g** Pretreatment of primary mouse astrocytes with siRNA of σ-1R, JNK, or p38 significantly inhibited LPS-mediated translocation of FOXJ2 into the nucleus. All the data are presented as mean ± SEM of three individual experiments. **p < 0.01 vs. the control group; #*p* < 0.05 and ##*p* < 0.01 vs. the LPS-treated control group

Next, we predicted HECTD1 transcription factor using GeneCards website and a candidate transcription factor, FOXJ2, attracts our attention (Fig. 6a). There were putative FOXJ2 binding sites within the sequence upstream of the HECTD1 promoter. In order to examine whether FOXJ2 physically binds to the upstream sequence of HECTD1 promoter, ChIP assays were performed. Intriguingly, LPS treatment resulted in increased binding of FOXJ2 to the HECTD1 promoter, thereby suggesting a putative regulatory element within the binding site (Fig. 6b). In addition, LPS increased the expression of FOXJ2 in the whole cell lysates with a concomitant increase in translocation of FOXJ2 into the nucleus (maximal response within 5 min) (Fig. 6c, d). LPS exposure also increased the expression of FOXJ2 in the cytoplasmic fraction (Fig. 6e).

Then we examined whether there exists a link that could tie together the activation of σ-1R-JNK/p38 pathway and FOXJ2 translocation into nucleus. Primary mouse astrocytes were thus pretreated with BD1047, SP600125, or SB203580 followed by treatment with LPS. As shown in Fig. 6f, pretreatment with all the inhibitors resulted in inhibition of translocation of FOXJ2 into the nucleus. To further confirm the observation, we applied siRNA of σ-1R, JNK, and p38, and found that knockdown of σ-1R, JNK, or p38 prevent the translocation of FOXJ2 into the nucleus (Fig. 6g). These findings linked LPS-mediated activation of σ-1R-JNK/p38 to downstream translocation of FOXJ2.

## Discussion

Our study provides new insights into the function of HECTD1 in astrocyte activation induced by LPS, indicating that HECTD1 may be a potential therapeutic target for the treatment of inflammatory diseases in CNS.

HECTD1 is an E3 ubiquitin ligase and its physiological function is mainly depend on the target proteins it binds to. Ubiquitin ligase E3 recognizes target proteins specifically, plays an important role in the ubiquitin pathway, and is involved in a variety of cellular physiological processes by regulating the ubiquitination of proteins [18]. Our study clarified that HECTD1 participates in astrocyte activation induced by LPS. Interestingly, HECTD1 has opposite effects on astrocyte activation induced by 100 ng/ml or 1 μg/ml LPS. A few studies have displayed contradictory evidences of HECTD1-medicated effects on cell migration. Li X found that HECTD1 ubiquitinated phosphatidylinositol 4-phosphate 5-kinase type I (PIPKI) γ90 and caused its degradation, and thus regulated migration of breast cancer cells. Suppressing the ubiquitination of PIPKIγ90 medicated by HECTD1 regulated the directionality of cell migration [19]. However, another recent study showed that SiO_2_-induced increases in cell proliferation and migration were restored by overexpression of HECTD1 via the CRISPR/Cas9 system, implying that HECTD1 inhibits cell migration [20]. Consistent with the study of SiO_2_-induced migration, detailed examination of cell motion on MEF cells suggested that lack of HECTD1 resulted in accelerated cell spreading and migration but impaired directionality of migration [21]. In addition to cell migration, it is possible for HECTD1 to play dual role in certain cellular processes, like regulation of astrocyte activation, by interacting with different ubiquitination substrate. Our study provides convincing evidences that HECTD1 is involved in LPS-mediated astrocyte activation. Our results manifest that downregulating the expression of HECTD1 inhibited activation of primary mouse astrocytes and HECTD1 overexpression enhanced astrocyte activation induced by 100 ng/ml LPS. However, HECTD1 showed opposite effect on astrocyte activation treated with 1 μg/ml LPS. The reason resulting in the difference is likely that disparate proteins are involved in the HECTD1-mediated ubiquitination process.

This is the first study of HECTD1 in astrocyte activation implicating that HECTD1 links with astrocyte-mediated inflammation induced by LPS. In our previous study, we found that circDLGAP4 acts as an endogenous miR-143 sponge to inhibit miR-143 activity, resulting in increased expression of HECTD1 and restored neuronal function [22]. What’s more, it is discovered that circDYM functions as an endogenous miR-9 sponge to suppress miR-9 activity, resulting in increased expression of HECTD1 and elevated HSP90 ubiquitination, thus inhibits microglia activation [14]. Our findings indicate that HECTD1 can regulate LPS-induced astrocyte activation. Taken together, HECTD1 may play different roles in various cell types-medicated inflammation via different ubiquitination substrate.

This study underscores the involvement of σ-1R-JNK/p38-FOXJ2 cascade in LPS-mediated induction of HECTD1 and GFAP in astrocytes. In this study, σ-1R antagonist (BD1047) blocked the activation of p38 MAPK and inhibited LPS-induced astrocyte activation. These data lend credence to previous report that administration of BD1047 significantly decreased the level of p-p38 expression induced by chronic constriction injury (CCI) in GFAP-labelled cells and attenuated the CCI-induced increase in GFAP expression [23]. In the current study, we demonstrated that LPS exposure increased GFAP in astrocytes via the p38 MAPK pathway, and the study by Ashley had similar finding that LPS-induced expression of cyclooxygenase 2 (COX2) decreased in response to inhibition of p38, but was unaffected by inhibition of MEK/Erk or JNK [24]. Additionally, our study is the first to demonstrate that FOXJ2 is involved in LPS-induced HECTD1 expression in primary mouse astrocytes. FOXJ2 is a member of Forkhead box transcription factors. Previous studies have found that FOXJ2 played an important role in LPS-induced inflammatory responses [25] and overexpression of FOXJ2 significantly inhibited migration of U87 cells [26]. Taken together, our results confirmed the significance of HECTD1 in LPS-induced activation of astrocyte and revealed that HECTD1 may serve as a potential target to develop new therapeutic agents for neuroinflammatory disorders.

## Conclusions

**Fig. 7:**
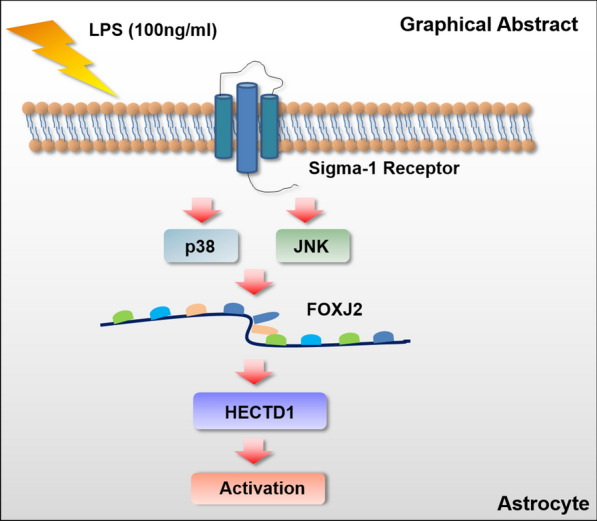
Schematic of the signaling pathways involved in LPS-mediated astrocyte activation. Exposure of LPS leads to σ-1R-mediated activation of MAPKs signaling pathways, JNK/MAPK and p38/MAPK pathways, results in the subsequent activation of downstream FOXJ2 transcription factor, thus enhances HECTD1 expression and subsequently induces astrocyte activation

In general, our study elucidated the role of HECTD1 in LPS-induced activation of astrocytes, thereby providing insight into the potential use of HECTD1 to develop novel therapeutic strategies for CNS diseases (Fig. 7).

## Supplementary Information

**Additional file 1: Figure S1.** Role of HECTD1 in astrocyte activation induced by 1 μg/ml LPS. a, b Treatment of primary mouse astrocytes with 1 μg/ml LPS significantly increased the expression of GFAP and decreased the expression of HECTD1. Representative immunoblots and the densitometric analysis from three separate experiments are presented. **p < 0.01 and ***p < 0.001 vs. the control group. c, d Representative immunoblots and the densitometric analysis from three separate experiments showed the effect of HECTD1 NIC and ACT transfection with/without 1 μg/ml LPS treatment on GFAP and HECTD1 expression. Transfection with HECTD1 NIC enhanced LPS-induced GFAP expression (c). Transfection with HECTD1 ACT inhibited LPS-induced GFAP expression (d). *p < 0.05, **p < 0.01, and ***p < 0.001 vs. the control group; #*p* < 0.05, ##*p* < 0.01, and ###*p* < 0.001 vs. the LPS-treated control group. **Figure S2.** HECTD1 suppressed astrocyte activation induced by 1 μg/ml LPS. a, b Representative images of HECTD1 fluorescence (green) and GFAP fluorescence (red) showed the effect of HECTD1 NIC and ACT transfection with/without 1 μg/ml LPS treatment on GFAP and HECTD1 expression. Scale bar=50 μm. Quantification of GFAP and HECTD1 immunofluorescence intensity used Image J software. *p < 0.05, **p < 0.01, and ***p < 0.001 vs. the control group; #*p* < 0.05 and ##*p* < 0.01 vs. the LPS-treated control group.

## Data Availability

The datasets used and/or analyzed during the current study are available from the corresponding author on reasonable request.

## Abbreviations

CCI: Chronic constriction injury
ChIP: Chromatin Immunoprecipitation
CNS: Central nervous system
COX2: Cyclooxygenase 2
FOXJ2: Forkhead box J2
GFAP: Glial fibrillary acidic protein
HECTD1: HECT domain E3 ubiquitin protein ligase 1
LPS: Lipopolysaccharide
PIPKI: Phosphatidylinositol 4-phosphate 5-kinase type I
SEM: Standard error of the mean
σ-1R: σ-1 Receptor
