# Supplementary material for: Involvement of HECTD1 in LPS-induced astrocyte activation via σ-1R-JNK/p38-FOXJ2 axis
Source: Cell Biosci. 2021 Mar 30;11:62. doi: 10.1186/s13578-021-00572-x (PMC8008527; doi:10.1186/s13578-021-00572-x)
Supplement: Supplementary file 1 — Additional file 1: Figure S1. Role of HECTD1 in astrocyte activation induced by 1 μg/ml LPS. a, b Treatment of primary mouse astrocytes with 1 μg/ml LPS significantly increased the expression of GFAP and decreased the expression of HECTD1. Representative immunoblots and the densitometric analysis from three separate experiments are presented. **p < 0.01 and ***p < 0.001 vs. the control group. c, d Representative immunoblots and the densitometric analysis from three separate experiments showed the effect of HECTD1 NIC and ACT transfection with/without 1 μg/ml LPS treatment on GFAP and HECTD1 expression. Transfection with HECTD1 NIC enhanced LPS-induced GFAP expression (c). Transfection with HECTD1 ACT inhibited LPS-induced GFAP expression (d). *p < 0.05, **p < 0.01, and ***p < 0.001 vs. the control group; #p < 0.05, ##p < 0.01, and ###p < 0.001 vs. the LPS-treated control group. Figure S2. HECTD1 suppressed astrocyte activation induced by 1 μg/ml LPS. a, b Representative images of HECTD1 fluorescence (green) and GFAP fluorescence (red) showed the effect of HECTD1 NIC and ACT transfection with/without 1 μg/ml LPS treatment on GFAP and HECTD1 expression. Scale bar=50 μm. Quantification of GFAP and HECTD1 immunofluorescence intensity used Image J software. *p < 0.05, **p < 0.01, and ***p < 0.001 vs. the control group; #p < 0.05 and ##p < 0.01 vs. the LPS-treated control group. [file 13578_2021_572_MOESM1_ESM.docx]

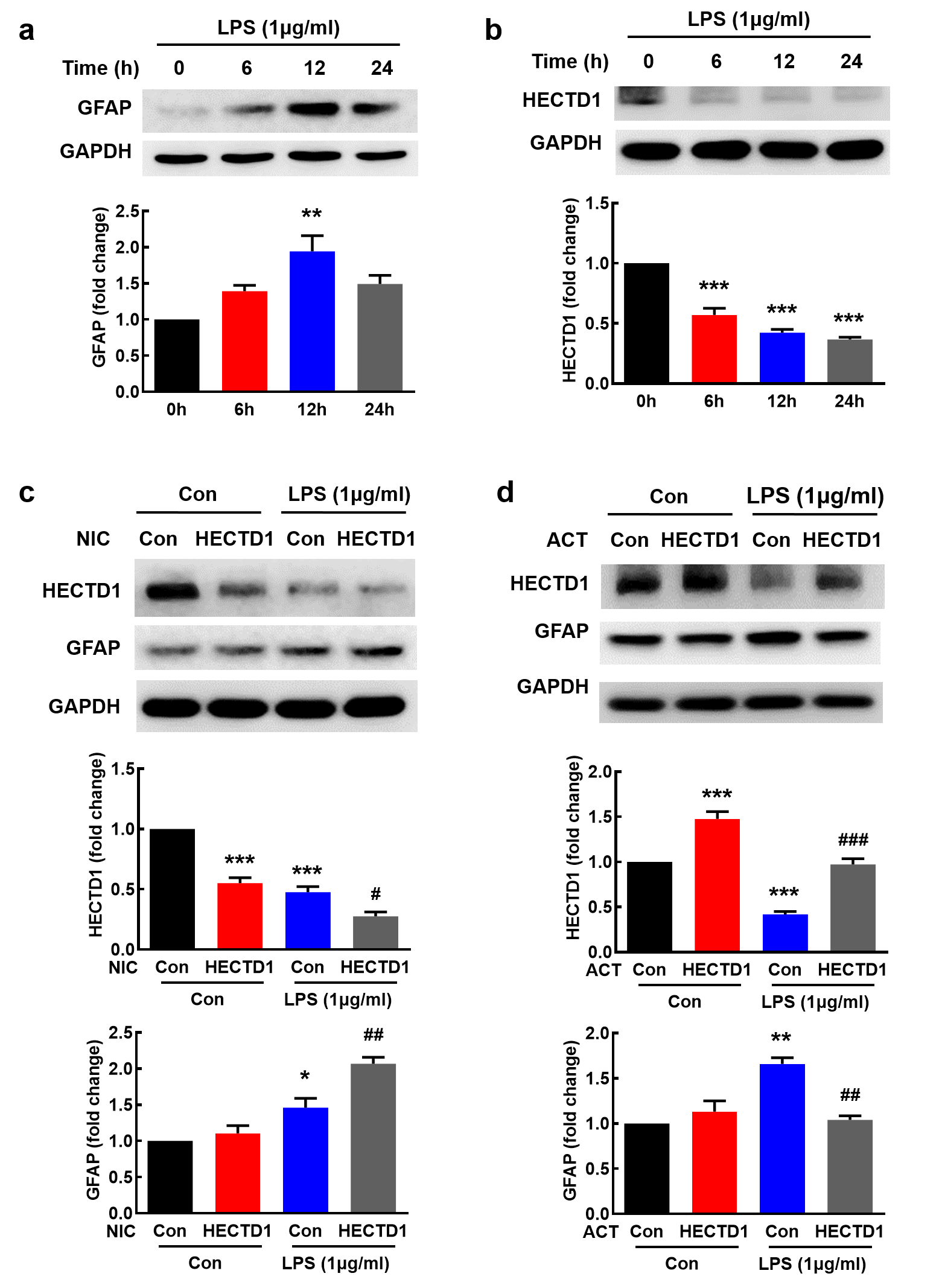


**Supplementary fig. 1** Role of HECTD1 in astrocyte activation induced by 1 μg/ml LPS. **a**, **b** Treatment of primary mouse astrocytes with 1 μg/ml LPS significantly increased the expression of GFAP and decreased the expression of HECTD1. Representative immunoblots and the densitometric analysis from three separate experiments are presented. ***p*<0.01 and ****p*<0.001 *vs*. the control group. **c**, **d** Representative immunoblots and the densitometric analysis from three separate experiments showed the effect of HECTD1 NIC and ACT transfection with/without 1 μg/ml LPS treatment on GFAP and HECTD1 expression. Transfection with HECTD1 NIC enhanced LPS-induced GFAP expression (**c**). Transfection with HECTD1 ACT inhibited LPS-induced GFAP expression (**d**). **p*<0.05, ***p*<0.01, and ****p*<0.001 *vs*. the control group; #*p*<0.05, ##*p*<0.01, and ###*p*<0.001 *vs*. the LPS-treated control group.


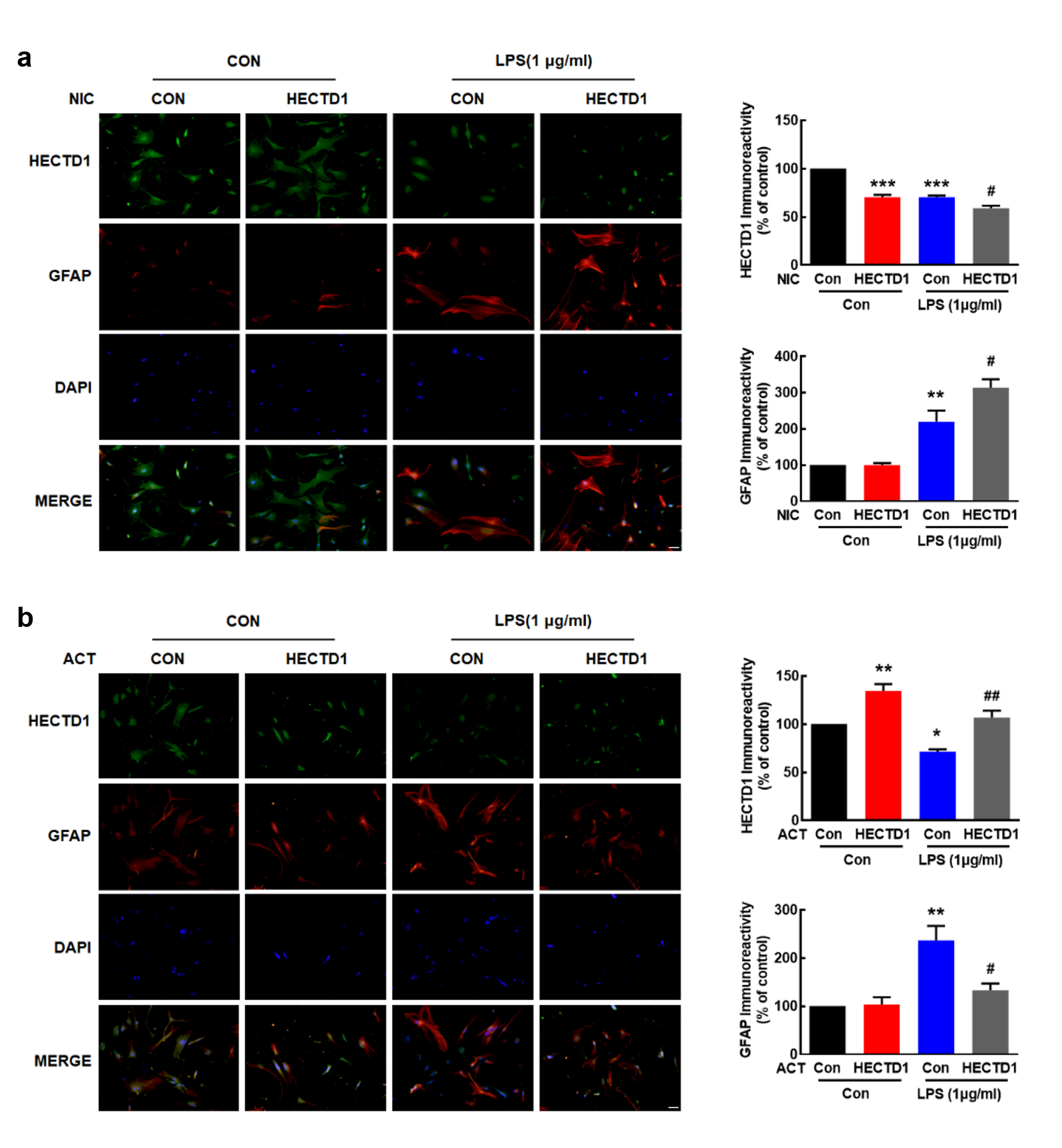


**Supplementary fig. 2** HECTD1 suppressed astrocyte activation induced by 1 μg/ml LPS. **a**, **b** Representative images of HECTD1 fluorescence (green) and GFAP fluorescence (red) showed the effect of HECTD1 NIC and ACT transfection with/without 1 μg/ml LPS treatment on GFAP and HECTD1 expression. Scale bar=50 μm. Quantification of GFAP and HECTD1 immunofluorescence intensity used Image J software. **p*<0.05, ***p*<0.01, and ****p*<0.001 *vs*. the control group; #p<0.05 and ##*p*<0.01 *vs*. the LPS-treated control group.
